# Telomere Dysfunction in Idiopathic Pulmonary Fibrosis

**DOI:** 10.3389/fmed.2021.739810

**Published:** 2021-11-11

**Authors:** Kexiong Zhang, Lu Xu, Yu-Sheng Cong

**Affiliations:** Key Laboratory of Aging and Cancer Biology of Zhejiang Province, School of Basic Medical Sciences, Hangzhou Normal University, Hangzhou, China

**Keywords:** telomere dysfunction, telomere shortening, alveolar stem cells, SASP, innate immune cells, TGF-β

## Abstract

Idiopathic pulmonary fibrosis is an age-dependent progressive and fatal lung disease of unknown etiology, which is characterized by the excessive accumulation of extracellular matrix inside the interstitial layer of the lung parenchyma that leads to abnormal scar architecture and compromised lung function capacity. Recent genetic studies have attributed the pathological genes or genetic mutations associated with familial idiopathic pulmonary fibrosis (IPF) and sporadic IPF to telomere-related components, suggesting that telomere dysfunction is an important determinant of this disease. In this study, we summarized recent advances in our understanding of how telomere dysfunction drives IPF genesis. We highlighted the key role of alveolar stem cell dysfunction caused by telomere shortening or telomere uncapping, which bridged the gap between telomere abnormalities and fibrotic lung pathology. We emphasized that senescence-associated secretory phenotypes, innate immune cell infiltration, and/or inflammation downstream of lung stem cell dysfunction influenced the native microenvironment and local cell signals, including increased transforming growth factor-beta (TGF-β) signaling in the lung, to induce pro-fibrotic conditions. In addition, the failed regeneration of new alveoli due to alveolar stem cell dysfunction might expose lung cells to elevated mechanical tension, which could activate the TGF-β signaling loop to promote the fibrotic process, especially in a periphery-to-center pattern as seen in IPF patients. Understanding the telomere-related molecular and pathophysiological mechanisms of IPF would provide new insights into IPF etiology and therapeutic strategies for this fatal disease.

## Introduction

Idiopathic pulmonary fibrosis is a progressive and fatal lung disease of unknown etiology. The pathological manifestation of this disease is the deposition of excessive extracellular matrix filaments in the interstitial space and gradual loss of alveolar epithelial cells in the lung parenchyma (1, 2). Once diagnosed, the average survival time is 3–5 years, and patients usually die of respiratory failure. At present, no efficient treatment options are available for this disease, and mortality is increasing every year (3). Recent progress has revealed that telomere dysfunction drives idiopathic pulmonary fibrosis (IPF) genesis. Understanding the role of telomere dysfunction in IPF would provide new insights into the early-stage diagnosis and the effective therapeutic strategies for this fatal disease.

## Telomere Structure And Function And Cellular Senescence

**Figure 1:**
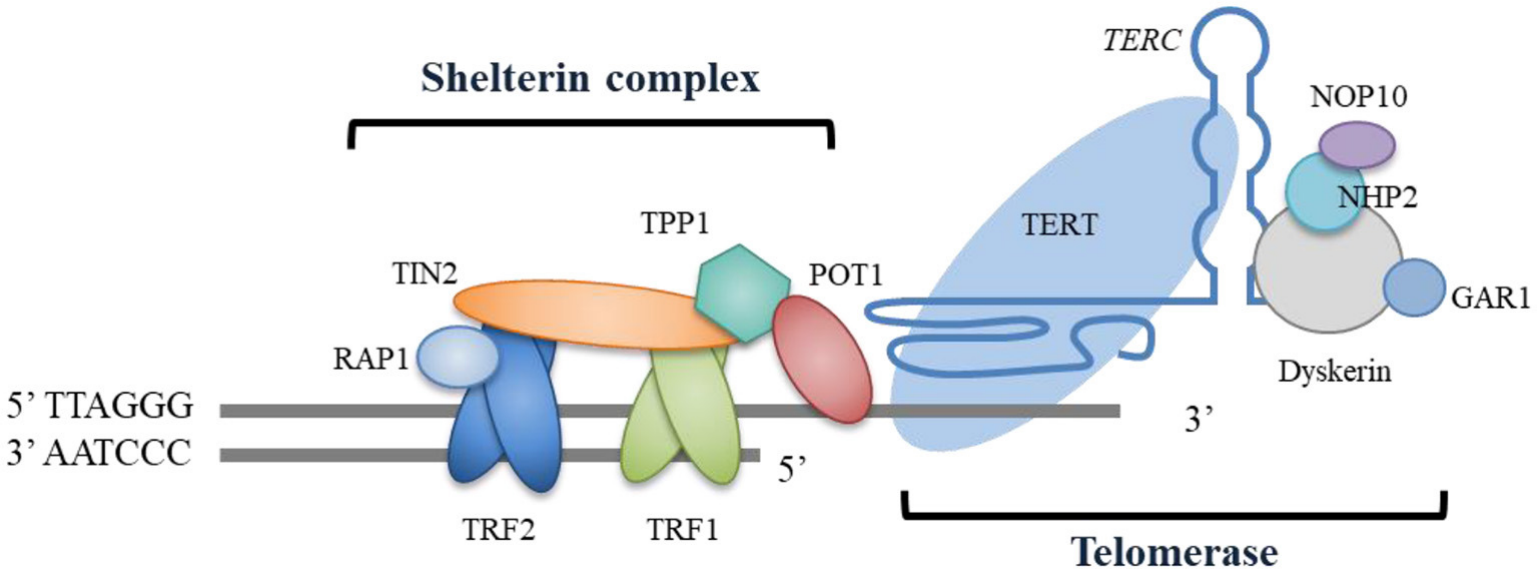
Schematic telomere structure and essential components. Human telomeric repeated DNA is protected by the shelterin complex, which consists of the protein components TRF1, TRF2, RAP1, TIN2, TPP1, and POT1. The ends of telomeres are bonded with telomerase, which is comprised of the catalytic subunit of TERT and the telomerase RNA component of *TERC*, and this exerts its effects by adding telomeric sequences to the telomeric ends. The telomerase also has other accessory components, including dyskerin, NHP2, NOP10, and GAR1. The function of these accessory components is to stabilize the *TERC*.

Telomeres are ribonucleoprotein structural complexes located at eukaryotic chromosomal ends and are mainly composed of tandem DNA repeats (TTAGGG in humans) and their binding proteins. Telomeres protect genome integrity and prevent degradation and chromosomal end-to-end fusion (4). Telomeric DNA usually terminates with a single-stranded 3′ G-rich overhang, which folds back to form a *t*-loop lariat structure with telomeric double-stranded repeats (5). This structure of telomeric DNA ends is capped and protected by a specialized shelterin complex that comprises six protein subunits (4, 5) (Figure 1). The ablation or deficiency of any shelterin component can result in telomere dysfunction, which triggers a DNA damage response. In most somatic cells, the telomere shortens at each cell division due to the end replication problem. However, stem cells, including adult stem cells in organs, express telomerase to counteract this shortening by adding telomeric sequences to the ends. Telomerase mainly consists of the catalytic subunit telomerase reverse transcriptase (TERT) and telomerase RNA component (TERC). It also has other accessory components, such as the most characterized element dyskerin, which combines with the other three smaller proteins NHP2, NOP10, and GAR1 to stabilize the TERC (6) (Figure 1). Additional components have also been identified as involved in telomerase assembly, trafficking, and regulation (6–8). In humans, telomere length is negatively correlated with age. When shortened telomeres reach a critical threshold, cellular senescence, and/or apoptosis are triggered by the DNA damage response (9).

Cellular senescence, first described by Hayflick, is a cellular phenomenon of stable cell cycle arrest characterized by a series of dramatic changes, including those in cell morphology, metabolism, epigenome, and gene expression (10). Although cellular senescence is a state of proliferation arrest, senescent cells are still metabolically active, producing a distinct secretome with a variety of cytokines and chemokines, known as the senescence-associated secretory phenotype (SASP) (10–13). Cellular senescence can be triggered by telomere shortening, certain active oncogenes, oxidase stress, and embryonic developmental signals (10, 11, 13–15). Accumulating evidence suggests that cellular senescence plays a critical role in aging-associated diseases (10, 16, 17). In the lung tissue, several studies suggest that the cellular senescence of alveolar epithelial type 2 cells (AEC2s) is involved in the genesis of IPF (18–20).

## Telomere Biology Disorders And Chronic Lung Diseases

Telomere biology disorders are a heterogeneous phenotypic group of illnesses arising from germline mutations that affect telomere maintenance (21). The first telomere biology disorder (TBD) was described as dyskeratosis congenita (DC), a hereditary disorder seen in childhood, in which ~20% of DC patients develop pulmonary fibrosis in later life (21). DC-linked mutations in telomere genes include those of *DKC1, TINF2, NOP10, NHP2, TCAB1*, and *CTC1* (6). IPF is the most common manifestation of other adult TBDs (21). It is not surprising that more than one TBD or clinical presentation can be observed in the same patient or family, but usually, one disease is predominant (6, 21, 22). This might be caused by germline telomere gene mutations that can affect the homeostasis of multiple organ systems.

To date, mutations in many telomere-related genes have been found to be associated with IPF (Table 1) (23–43). Other telomere-related chronic lung diseases or phenotypes include chronic obstructive pulmonary disease (COPD), emphysema, chronic hypersensitivity pneumonitis, and rheumatoid arthritis-associated interstitial lung disease (44–48). It has been found that IPF and emphysema co-exist in the same pedigree, and IPF and COPD or lung cancer can co-occur in the same patient (39, 46, 49, 50).

**Table 1:** Gene mutations associated with telomere dysfunction in idiopathic pulmonary fibrosis.

**Gene**	**Product**	**Function**	**Telomere shortening**	**Mutation consequence**	**References**
*TERT*	TERT	Telomerase catalytic subunit	Y	Decreased telomerase activity, haploinsufficiency	(23–30)
*TERC*	TERC	Telomerase RNA component	Y	Decreased telomerase activity	(23–26, 30)
*DKC1*	DKC1	Dyskerin		Destabilized and decreased TERC, dyskerin deficiency	(31, 32)
*TINF2*	TIN2	Component of the shelterin complex	Y, N	Decreased TIN2, and telomere uncapping?	(33, 34)
*RTEL1*	RTEL1	DNA helicase in telomere maintenance	Y	Affect the function and/or stability of RTEL1, haploinsufficiency	(29, 30, 35, 36)
*PARN*	PARN	Exoribonuclease, TERC maturation	Y	Decreased TERC, haploinsufficiency?	(29, 30, 36–38)
*NAF1*	NAF1	Stabilizes TERC	Y	Low TERC level, haploinsufficiency	(39)
*NOP10*	NOP10	Stabilizes TERC	Y	Affect the stability of TERC?	(40)
*NHP2*	NHP2	Stabilizes TERC	?	?	(41)
*ACD*	TPP1	Telomerase recruitment to telomere	?	?	(42)
*ZCCHC8*	ZCCHC8	Mediating TERC 3' end targeting	Y	TERC insufficiency	(43)

*Gene name, coding protein and its function, and mutations associated with telomere dysfunction in IPF. Y, telomere shortening; N, no telomere shortening; ?, unknown*.

Accumulating evidence has implicated telomere dysfunction in the pathogenesis of IPF. However, the underlying molecular mechanisms remain unclear. Available data suggest that cellular senescence or death of alveolar stem cells induced by telomere dysfunction is implicated in pulmonary fibrosis by altering cell signaling and the microenvironment in the lung.

## Association Between Ipf And Telomere Abnormalities

It has been noticed that some IPF cases seem to be inherited, which was inferred from the observation of familial clustering of the disease and twin studies. Most IPF families display an autosomal dominant inheritance pattern with incomplete penetrance, suggesting that a single gene is responsible for disease pathogenesis in each family. A breakthrough discovery using linkage analysis identified *TERT* mutations in two IPF families (23). Subsequent sequence screening revealed that 15% of IPF families had *TERT* or *TERC* mutations, whereas 2% of sporadic IPF cases possessed *TERT* or *TERC* mutations. Patients with these heterozygous mutations in *TERT* or *TERC* had significantly shorter telomeres and decreased telomerase activity. A similar study screened 73 probands from the Vanderbilt Familial Pulmonary Fibrosis Registry and found that six (8%) had heterozygous mutations in *TERT* or *TERC* (24). In a study of familial IPF with early-onset, it was found that some cases with mutations in TERT and markedly short telomeres present with extrapulmonary features, including liver cirrhosis, bone marrow hypoplasia, and premature graying (51–53). Intriguingly, overall, as many as 37% of familial IPF and 25% of sporadic IPF cases have shorter telomeres, whereas as many as 24% of familial IPF and 23% of sporadic IPF cases without *TERT* or *TERC* mutations also have significantly short telomeres, suggesting the existence of other gene mutations in IPF (25, 26). Indeed, many telomere gene mutations have been associated with IPF (Table 1) (23–43). Together, these studies identified telomere dysfunction as a major driver of IPF.

## Alveolar Stem-Cell Failure Induced By Telomere Dysfunction

Genetic studies on IPF have indicated that ~40% of familial IPF and 25% of sporadic IPF cases have significant telomere shortening and that 15% of familial IPF is due to *TERT* or *TERC* mutations. This fact suggests the importance of telomere dysfunction in IPF pathogenesis, but the question of how telomere dysfunction drives the genesis of pulmonary fibrosis remains. The capacity to regenerate lung tissue was determined to be significantly decreased in generation (G) 4 *TERC* KO mice after partial pneumonectomy, indicating that adult lung stem cells are significantly affected by telomere shortening (54). Moreover, in these G4 *TERC* KO mice, the number of AEC2s was significantly decreased and the apoptotic pathway was activated in the lungs, suggesting that AEC2s are vulnerable to apoptosis during telomere shortening (55). Interestingly, we observed that AEC2s in G3 *TERC* KO mice present with significant cellular senescence (56, 57). In addition, we found that G2 *TERC* KO mice display aggravated bleomycin (BLM)-induced pulmonary fibrosis, suggesting that telomere shortening promotes the genesis of pulmonary fibrosis (57). In line with this observation, late-generation *TERT* KO mice develop increased pulmonary fibrosis under low-dose BLM induction (58). Furthermore, spontaneous pulmonary fibrosis was found to occur after conditionally deleting the telomere shelterin component telomeric repeat binding-factor (TRF1) in AEC2s (58, 59). In *TRF1* conditional KO fibrotic lungs, the loss of AEC2 (~2/3), and increased lung cellular senescence and apoptosis were observed. Thus, these findings suggest the important role of alveolar stem cell AEC2s in the pathogenesis of pulmonary fibrosis (58–60). However, pulmonary fibrosis has not been observed in a mouse model of *TRF1* deletion in fibroblasts, suggesting that telomere dysfunction leading to pulmonary fibrosis might be dependent on AEC2s (59). Interestingly, in conditional *TRF1* KO mice, the telomere lengths of AEC2s are normal at the early stage, whereas marked telomere shortening was observed at the late stage, suggesting that the long-term deletion of *TRF1* might lead to telomere shortening within AEC2s (59). Similarly, AEC2-conditional *TRF2* KO mice exhibit significant telomere dysfunction and cellular senescence (61). Moreover, a recent study suggested that TPP1 degradation leads to telomere uncapping in AEC2s, thereby promoting stress-induced cellular senescence and pulmonary fibrosis (62). An investigation of IPF with or without TERT mutations indicated that AEC2s, but not other surrounding cells, have significantly short telomeres (63). These studies imply that telomere dysfunction might preferably affect lung AEC2s and compromise these cells to generate spontaneous pulmonary fibrosis.

The animal models discussed above suggest that AEC2 failure plays a vital role in the development of pulmonary fibrosis. However, it is unclear how dysfunctional AEC2s cause the fibrotic process. One possibility might be due to defective AEC2 signaling other cells (61). Recently, a transgenic animal model study suggested that the cellular senescence, but not apoptosis, of AEC2s promotes the development of pulmonary fibrosis (19). A recent interesting discovery from a mouse model showed that conditionally deleted Cdc42 in AEC2s leads to progressive pulmonary fibrosis in mice, as seen in IPF patients (64). This study demonstrated that the deletion of Cdc42 results in the impaired differentiation ability of AEC2s to AEC1s, which suppresses new alveoli formation, in turn increasing mechanical tension in the lungs with a non-uniform distribution (64). Given the deficiency in the stem cell functions of AEC2s observed in late-generation *TERC* KO mice and conditional *TRF2*-deleted mice, it is possible that telomere dysfunction might be associated with this mechanical tension mechanism to promote pulmonary fibrosis caused by the inability to form new alveoli.

## The Senescence-Associated Secretory Phenotype And Innate Immune Cell Infiltration Induce A Pro-Fibrotic Niche In The Lung

Senescent cells are metabolically active and produce a distinct SASP with various cytokines, chemokines, growth factors, and matrix metalloproteinases (10–13). Transforming growth factor-beta (TGF-β) secreted from senescent cells was found to cause senescence in neighboring cells, which might be important for organ aging, particularly in slowly self-renewing tissues such as the lung, because AEC2 cellular senescence might spread to other cells within the same organ (13). In an animal model, the deletion of *TRF1* in AEC2s was found to result in the senescence of AEC2s and elevated levels of lung TGF-β1 (59). Similarly, lungs from late-generation (G2 and G3) *TERC* KO mice show significantly increased TGF-β1 and activated TGF-β/Smad signaling (57). Consistently, single-cell RNA-sequencing analysis revealed that IPF lungs harbor senescent AEC2s with increased TGF-β1 in these cells (65). In addition, microarray analysis of AEC2 TRF2-deficient mice revealed that the upregulated genes are involved in immune signaling and inflammation pathways (61). Moreover, in the late generation of both *TERC* and *TERT* KO mouse lungs, remarkably elevated levels of various cytokines, including IL-1, IL-6, CXCL15, IL-10, TNF-α, and CCL2, have been noted (56, 66).

It is widely observed that the lungs from transgenic mice with telomere dysfunction show innate immune cell infiltration, accompanied by lung inflammation. Late-generation *TERC*-deficient mouse lungs have increased CD11b^+^, CD16/CD32^+^, or CD45^+^ cells, macrophages, neutrophils, and natural killer (NK) cells (56, 57). *TRF1*-deficient mice show extensive macrophage infiltration in the lungs, intense pneumonia, and increased numbers of mononuclear cells, neutrophils, and lymphocytes in bronchoalveolar lavage (BAL) fluids (58, 59). Similarly, in conditional *TRF2* KO mice, the BAL fluids contain a significantly increased number of macrophages and lymphocytes and the lungs exhibit macrophage inflammatory infiltrates and peribronchiolar and perivascular inflammation (61). These data suggest that telomere dysfunction promotes significant infiltration or the recruitment of innate immune cells in the lungs. In addition to its role in the phagocytosis of senescent and apoptotic cells, macrophages play a promoting role in the development of pulmonary fibrosis (67). Several profibrotic genes such as *arginase1* and *matrix metallopeptidase 13* (*MMP13*) were found to be upregulated in monocyte-derived macrophages in experimental pulmonary fibrosis, as well as in resident alveolar macrophages in IPF patients (68). Moreover, M2 macrophages are an important regulator of pulmonary fibrosis, partially through the secretion of profibrotic interleukin-10 (IL-10) and TGF-β (67). Whether and how the recruitment of macrophages in the lungs caused by telomere dysfunction participates in pulmonary fibrosis development requires further investigation (69).

**Figure 2:**
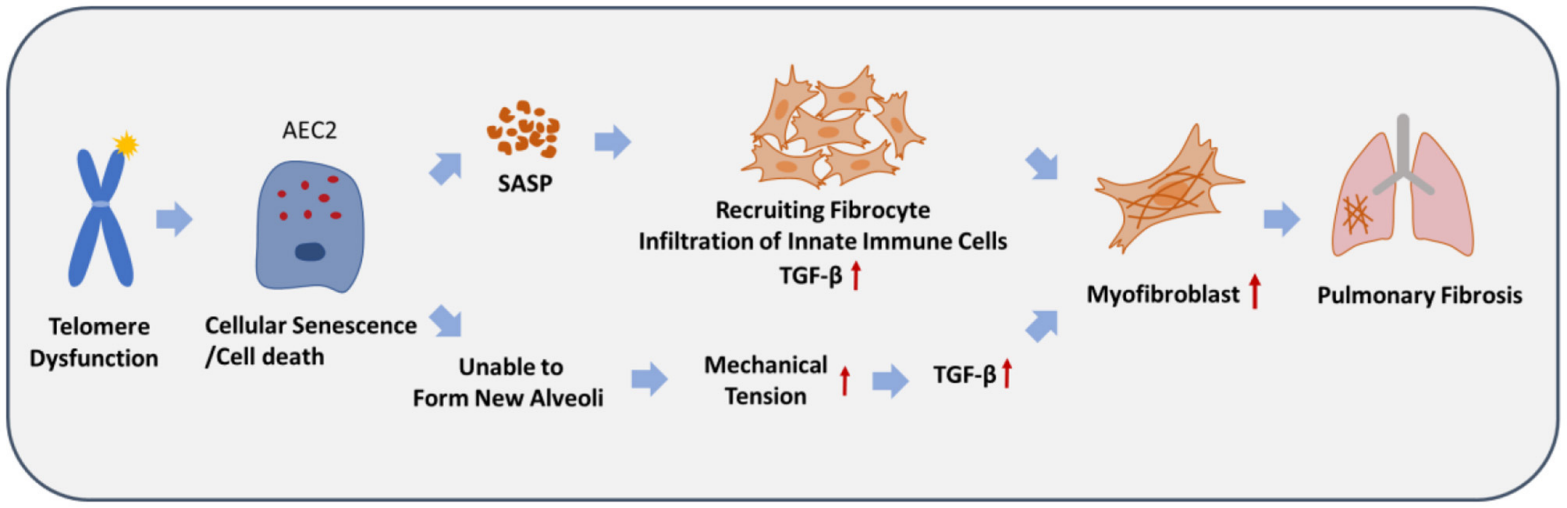
A proposed model to depict the pathogenesis of idiopathic pulmonary fibrosis caused by telomere dysfunction. Telomere dysfunction causes cellular senescence and/or cell death of alveolar epithelial type 2 cells (AEC2s), which leads to the production of a pro-fibrotic niche, mediated by the SASP. This probably leads to increased mechanical tension and a TGF-β signaling loop in a spatially distributed manner in the lung, owing to the inability to form new alveoli. Eventually, pulmonary fibrosis develops due to the significant increase in myofibroblasts.

In summary, we propose a model to demonstrate how telomere dysfunction drives the pathogenesis of IPF (Figure 2). Telomere-related mutations or telomere shortening preferentially affect alveolar stem cell AEC2s in the lung and cause senescence or apoptosis. This cellular dysfunction is sufficient to generate spontaneous pulmonary fibrosis, mainly through two pathways. First, cellular senescence and/or cell death of AEC2s leads to a pro-fibrotic niche through the SASP, and fibrocytes capable of differentiating into fibroblasts, myofibroblasts, and innate immune cells are recruited to the fibrotic lesion site. Second, the failure of AEC2s compromises the regeneration of new alveoli, which in turn leads to an increase in mechanical tension in a periphery-to-center spatial distribution, which activates the TGF-β signaling loop in AEC2s or other lung cells. These two pathways might significantly increase the local levels of TGF-β, myofibroblast differentiation, and fibrotic lesions in the lung tissue.

## Discussion

The available data from human IPF cases and animal model studies have established a connection between telomere dysfunction and pulmonary fibrosis mediated by alveolar stem cell AEC2s. However, it is still unclear how these cells promote the development of pulmonary fibrosis. Although some animal model studies support the idea that cellular senescence of AEC2s drives the development of pulmonary fibrosis (19, 59, 61), other animal studies suggest that AEC2 cell death plays an important role in the development of pulmonary fibrosis (58). It is likely that senescent AEC2s signal other cells through the SASP (61). Apoptotic, necrotic, necroptotic, and pyroptotic cells can also release diverse cytokines; specifically, apoptotic cells secrete IL-10 and TGF-β, necrotic cells release IL-1α, and necroptotic cells release IL-8, IL-1, chemokine ligand 2 (CXCL2), and cyclophilin A, whereas pyroptotic cells secrete IL-1β, IL-18, tumor necrosis factor-alpha (TNF-α), and IL-6 (70). The secretion of these factors is thought to lead to the recruitment of innate immune cells to remove these dying cells to regulate inflammation and wound healing, but the release of excessive amounts of IL-10 and TGF-β might contribute to pulmonary fibrosis. Thus, the contribution of cellular senescence and cell death of AEC2s to the pathogenesis of pulmonary fibrosis requires further investigation.

Although it is now well-recognized that telomere dysfunction preferably affects lung AEC2 function and is a major driver of pulmonary fibrosis, other stem cells such as club cells and basal cells might also be affected by telomere dysfunction, thereby exerting their respective effects. Notably, 40% of familial IPF and 25% of sporadic IPF cases have significantly short telomeres, in which genetic alterations in telomere-related genes have been identified, suggesting the important role of telomere dysfunction in the pathogenesis of pulmonary fibrosis. Furthermore, it has been reported that some telomere-unrelated genes are also implicated in the genesis of pulmonary fibrosis, suggesting complex mechanisms of IPF at different pathophysiological levels (71, 72).

It is worth mentioning that IPF cases are usually seen in old age, and some relatives of IPF patients with both *TERT* or *TERC* mutations and telomere shortening do not develop pulmonary fibrosis, suggesting that aging and environmental factors are also involved in disease pathogenesis (25). The aging-associated changes in telomere length, stem cell senescence, autophagy, endoplasmic reticulum stress responses, immunosenescence, and epigenetics, as well as cumulative environmental exposure, might contribute to disease development through an aging mechanism (73, 74). In addition, viral infection, cigarette smoking, and occupational exposure could also confer a risk of IPF (73, 75).

Clinical outcomes and therapeutic responses might differ based on different genotypes in IPF; further understanding this difference will allow for the development of personalized approaches to disease diagnosis, management, and treatment (76, 77). Patients with remarkably short telomeres have more rapid disease progression, a shorter lung transplant-free survival time, and a poor prognosis (78). It has been found that IPF patients with mutations in *TERT, PARN, TERC*, or *RTEL1* have shorter telomeres and earlier disease onset than patients without, and IPF cases with *TERC* mutations are diagnosed at an earlier age than those with *PARN* mutations (30). Moreover, it has been found that IPF with mutations in *TERT, RTEL1*, or *PARN* has a significantly higher risk of death and chronic lung allograft dysfunction compared to those in patients without these mutations (79, 80). Advances in next-generation sequencing and gene-chip technologies would facilitate the development of standard methods and criteria for the precise diagnosis of IPF. We anticipate that CRISPR/Cas9 genomic editing, stem cell therapy, telomerase treatment, or targeting cellular senescence will hold promise for the future treatment of this fatal disease.

## Author Contributions

KZ wrote the first draft of the manuscript. LX and KZ prepared the tables and figures. All authors critically revised the manuscript, reviewed the final version, agreed upon submission, and agree to be accountable for the content of the work.

## Funding

This work was supported by grants from the National Natural Science Foundation of China (31730020), Hangzhou Science and Technology Bureau (20182014B01), and Hangzhou Human Resources and Social Security Bureau (4125F5061700502).

## Conflict of Interest

The authors declare that the research was conducted in the absence of any commercial or financial relationships that could be construed as a potential conflict of interest.

## Publisher's Note

All claims expressed in this article are solely those of the authors and do not necessarily represent those of their affiliated organizations, or those of the publisher, the editors and the reviewers. Any product that may be evaluated in this article, or claim that may be made by its manufacturer, is not guaranteed or endorsed by the publisher.
